# Achieving universal health coverage in India: a scoping review on the requisite public health actions

**DOI:** 10.3389/fpubh.2024.1366355

**Published:** 2025-02-10

**Authors:** Avani Radheshyam, Vinod K. Ramani, Radheshyam Naik

**Affiliations:** ^1^Junior year, 10X International school, Bangalore, India; ^2^Department of Preventive Oncology, Healthcare Global Enterprise Ltd. (HCG), Bangalore, India; ^3^Department of Medical Oncology, Healthcare Global Enterprise Ltd. (HCG), Bangalore, India

**Keywords:** universal health coverage, insurance coverage, health policy, health equity, healthcare financing, India

## Abstract

**Introduction:**

In India, large inequities in health exist by geography, gender, socio-economic class, religion and caste. Universal health coverage (UHC) is envisioned to address these gaps. The deficiencies in our healthcare system cannot be solely bridged by additional investment, increasing manpower, adoption of technology or establishing regulatory Institutes. While UHC offers promise, its nation-wide implementation must be carefully planned and monitored.

**Objectives:**

(1) To review published literature appraising the concepts of UHC such as coverage of health services and financial protection in the Indian healthcare system, (2) To review the deficiencies of the healthcare system in India and explore solutions within the framework of UHC.

**Methods:**

The relevant articles for review were retrieved from PubMed and Google scholar databases using pertinent text terms. This scoping review includes 12 citations and 3 other published reports which address the determinants of UHC and its impact on the healthcare system in India.

**Results:**

UHC aims to address the concept of health in all its dimensions and not merely as a response to illness. This Program’s objectives include reducing the gap between the need and utilization of healthcare, improving its quality and providing financial protection. In India, the public health sector suffers from shortfalls in management, manpower issues and poor accountability, whereas the private health sector is unregulated and contributes to the increasing health expenditure. UHC will improve access to health care and prevent financial impoverishment, which will be advantageous to the rural people and urban poor including workers of the unorganized sector.

**Conclusion:**

UHC enables health systems to efficiently deliver a wide range of healthcare services to the population, as well as adopt sustainable financing mechanisms. Given the current Covid-19 pandemic and the need to address future pandemics, we need to build resilient health systems as well as hasten the implementation of UHC.

## Introduction

Universal health coverage (UHC) has been adopted as the United Nations (UN) Sustainable Development Goal (SDG) Target 3.8 (1). The concept is relevant to every Country, as the global priorities in health include equity in the utilization of health services, quality of care and financial protection of the community. The reforms in health sector should focus on making healthcare available and affordable to the socially and economically marginalized groups. The goal is to provide the whole population with a range of essential health services covering 100% of their healthcare needs, and ensuring they receive these services without incurring health expenditure. Currently, this expenditure accounts to >10% of the monthly household budget (2). Its emphasis is on prevention and primary health care, and also encompass building advocacy for the health needs of the community.

The WHO’s goal of ‘Health for all’ is reflected in the concept of ‘universal coverage’ (3). Under the Indian constitution, health is regarded as a State subject and every State/Union territory has developed its own healthcare delivery system based on the guidelines issued by the Government of India. The development of Indian health system is guided by the Bhore committee report of 1946: Jan Swasthya Abhiyan, Choosing Health report, and the Independent Commission on Health and Development report. These reports identify comprehensive primary health care as high priority, equity as the core value and community based approach as the key strategy (4). Lauer et al. (1) identify the three main indicators within the UHC framework:

- Coverage: Number of beneficiaries receiving the needed health services,
- Equity: Equitable distribution of access to health services across the population groups,
- Financial risk protection: People lacking power to purchase health services or become impoverished due to out-of-pocket payments,

Table 1 depicts the relevant data on certain sociodemographic and health parameters in India. During the period 2000 to 2017, the health expenditure of the Indian Government as a proportion of GDP grew from 0.83 to 0.96% (2). The current investment in health sector is ~1.2% of GDP, which on conversion equals USD 18 (INR 1350) per capita, much lesser than the average of 2.8% for other LMICs (2). In India, the total health expenditure is 4.7% of GDP (2) of which the share of private expenditure is high when compared with the public expenditure (3). Other pre-requisites for the delivery of UHC include the availability of skilled workforce, adequate healthcare infrastructure, and access to affordable drugs and technologies (5). In India, currently there are 0.65 Doctors, 1.3 Nurses and 1.3 hospital beds per 1,000 people (6). To address the morbidity of communicable and non-communicable diseases, we would require twice the number of Doctors, triple the number of nurses and quadruple the number of paramedic and support staff (6).

**Table 1:** Data on certain sociodemographic and health parameters of India.

Sl. No.	Parameter	Value
1	People per square kilometer (9)	382
2	Income of the highest earning member among ~75% of rural households^a^	< Rs.5,000/month (USD 67) (31)
3	Life expectancy^b^	68.7 years
4	Infant mortality rate^b^	33/1000 live births
5	Maternal mortality ratio^b^	130/100,000 live births
6	Total fertility rate^b^	2.3 children/woman
7	Private health sector	Provides service to ~70% of the population (6)
8	Financial risk protection of our population	17.9% (3)
9	Coverage for prevention and treatment of select health conditions	83.5% (3)

a Socio-economic and caste census 2011.

b National health profile 2019 (6).

Towards achieving its intended objectives, the National Health Policy of India (NHP)2017 (7) envisaged an increase in the central public health expenditure from the current 1.2 to 2.5% of the Gross Domestic Product (GDP) by the year 2025, and the state health sector spending to >8% of their budget by 2020 (3). The rising cost of healthcare in India is one of the leading causes of poverty (8), as the health system is primarily dependent on out-of-pocket (OOP) expenditure and accessing healthcare from the private providers. Annually, ~50 million households (20% of total Indian households) are estimated to fall below the poverty line due to OOP healthcare expenditure (9). The results of the impact evaluation of the previous public health insurance scheme RSBY [Rashtriya Swasthya Bhima Yojana; (8)] in India, shows a 30% increased likelihood of incurring OOP expenditure by the beneficiaries.

Kumar (10) opine that the implementation of National Health Mission (11) (NHM) in India since 2005, has enabled the development of public sector health systems based on a primary health care approach. Ayushman Bharat (AB) (12) scheme was launched in India during 2018, encompassing two complementary schemes: Health and Wellness centers (HWC), and National health protection scheme (PMJAY: Pradhan Mantri Jan Arogya Yojana) (3). HWC will result in improved health delivery across existing primary care centers which includes comprehensive primary care, free essential drugs and diagnostic services. This will enable the expansion of access, universality and equity of services in close proximity to the community. The target beneficiaries for PMJAY will be identified through the socio-economic caste census (13) database, which includes the vulnerable and marginalized households apart from the below poverty line (BPL) families (3). Health protection will cover ~500 million beneficiaries (bottom 40% of the Indian population), thus providing coverage upto INR 500,000 (equivalent to USD 6400) per family per year for secondary and tertiary care hospitalization (14) (including services from the private sector). AB-PMJAY (12) favors comprehensive outpatient care and services rather than just the hospitalization coverage as with the earlier RSBY scheme.

Our review follows the standard framework recommended by Arksey et al. (15).

### Framework stage 1: identifying the research question

In the Indian context, the major barriers to UHC include the low level of public investment in healthcare and engagement of the powerful private health sector. Until recently, there was lack of political commitment towards recognizing health as an essential component of human development. Investment in healthcare is perceived as a drain of resources rather than an outlay, unlike with the labor department which tends to drive the economic growth. This notion has led to low investment, lack of sound process for policy formulation and inappropriate health program implementation by the public sector, which in-turn results in inadequate delivery of health care measures. UHC is not merely an aspirational goal but an entitled provision (13). It ceases to be a system of health insurance but conceptually is an assurance of health care. The focus of our review is on various aspects of UHC which address health across the prevention to care continuum, and not merely as a response to illness.

### Objectives

(1) To review the published literature which appraise the concepts of UHC with regard to coverage of health services and financial protection in the Indian healthcare system, (2) To review the deficiencies of the healthcare system in India and explore solutions within the framework of UHC.

## Methods

### Framework stage 2: identifying relevant studies

We conducted a scoping review of PubMed and Google scholar databases during the period July to August 2022, using the following text terms with Boolean operators: ‘Universal health coverage’; ‘community health insurance’ and ‘Public health insurance in India’. The epistemology includes literature on UHC, encompassing 17 articles from PubMed and 8 grey literature (including reports) from Google scholar which incorporate a range of different methods and study designs. The review’s focus is to map the available evidence by explicating the relevant strategies of UHC from the selected literature, which subsequently contributes to the policy debate on UHC in India. It provides the range for identification of gaps in the literature and guidance for future research. The iterative nature of this review suits the exploratory research questions posed on the concepts of UHC. This qualitative approach includes the interpretivist and constructivist paradigms, thus enabling a reflection of perspectives and subjective experiences, respectively.

### Framework stage 3: study selection

The inclusion criteria for our review comprise of literature which illustrate the constructs of UHC, and discern the facilitators and barriers for its implementation. Scientific articles which do not address the determinants of UHC were excluded from the study. All the authors scrutinized the identified reviews, original articles and case studies which uniquely illustrate the relevant concepts, and the framework associated with UHC.

Author ‘AR’ teased the relevant literature to include 12 articles from ‘PubMed’ and 3 reports from ‘Google scholar’, which describe the concepts relevant to UHC. Authors ‘VR’ and ‘RN’ analyzed the screened articles for relevance to the defined review question, which includes discussing the characteristics of UHC, policy implications and path ahead for India. All the authors were involved with summarizing the data, synthesis of information regarding the programmatic implications of UHC and identifying the gaps in the Indian context.

## Results

### Framework stage 4: charting the data

**Figure 1:**
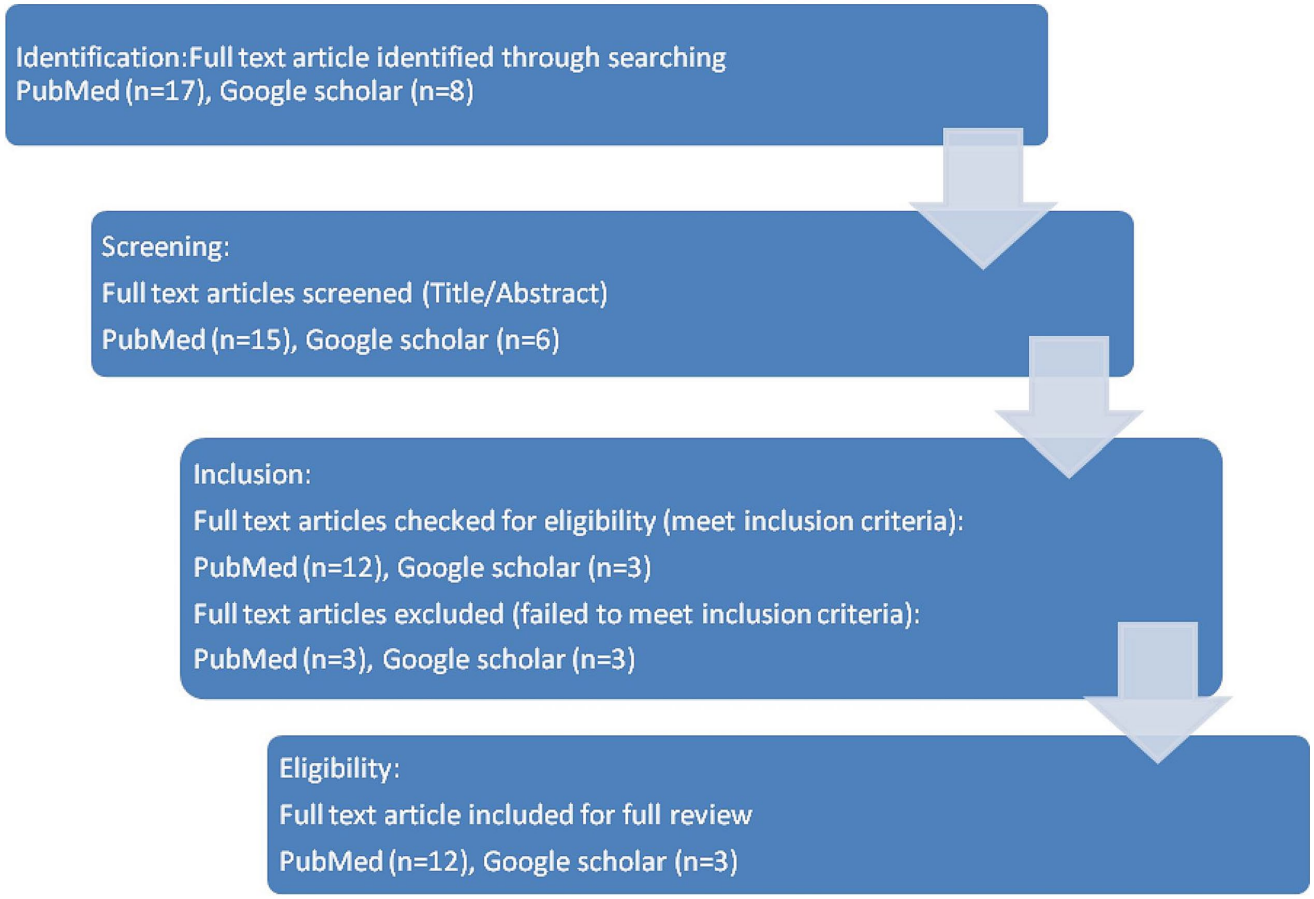
PRISMA four phase flow diagram of the process of article selection.

The results of our scoping review draws from 12 peer reviewed publications in Indian and international journals as well as 3 published reports. It adheres to the Preferred Reporting Items for Systematic Review and Meta-Analysis (PRISMA) extension for scoping reviews (PRISMA-ScR) (16). Figure 1 depicts the four phase flow diagram (as per PRISMA guidelines) illustrating the data charting process. The findings of the review are synthesized and presented under the guiding principles to aid the critical appreciation of gaps and the way forward for its implementation.

**Figure 2:**
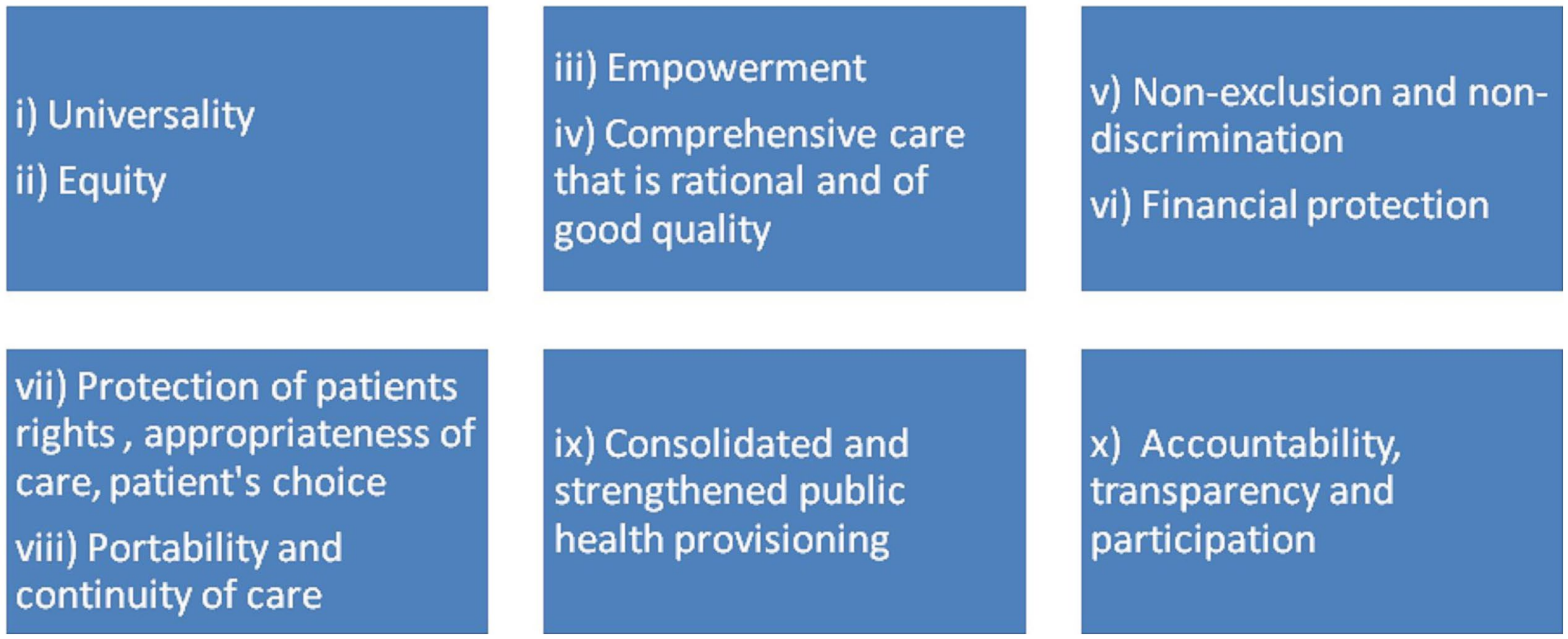
Ten principles guiding the formulation of UHC in India.

Figure 2 depicts the principles guiding the formulation of UHC in India. These are in consonance with the report drafted by the high level Expert group on UHC for India, which was submitted to the Planning Commission during November 2011 (17). The outcome contextualized in our narrative conforms to the process of these 10 principles.

Table 2 lists the details of published literature and their salient findings.

**Table 2:** Literature reviewed as part of the scoping review.

Sl. No.	Author and Title of article	Name of Journal and Year of publication	Salient findings
1	Lauer J.A et al. (1), Rajan D, Bertram M.Y. Priority setting for Universal health coverage: we need to focus both on substance and on process.	Int J Health Policy Manag 2017	Identify the three main indicators within the UHC framework: Coverage, Equity, Financial risk protection
2	Sundari T.K et al. (2). Towards Universal health coverage? Taking stock of two decades of health reforms in India.	India’s Economy and Society 2021	Evidence for PPPs’ which portray neither an increase in the available range of services nor a significant population-level impact on expanding coverage to the impoverished population
3	Zodpey S (6). Universal Health Coverage in India: Progress achieved & the way forward.	Indian J Med Res April 2018	The tax revenue in low and middle income countries (LMICs) is a key determinant in the progress towards UHC.
4	Reddy K.S et al. (31). Towards achievement of universal health care in India by 2020: a call to action	The Lancet 2011	Public spending on health should be increased from 1 to 6% of the GDP. This will enable reducing the proportion of OOP expenditure from 80 to 20%.
5	Kumar R (8). Achieving Universal Health Coverage in India: The Need for Multisectoral Public Health Action.	Indian J Community Med 2020	Propose the establishment of an autonomous Public health commission in India, which will enable achieving the coordination of initiatives such as health promotion, disease prevention and provision of health care.
6	High level Expert group (14) report on Universal health coverage for India.	www.niti.gov.in Nov 2011	Depicts the 10 principles guiding the formulation of UHC in India
7	Kutzin J (17). Health financing for universal coverage and health system performance: Concepts and implications for policy.	Bulletin of the World Health Organization Aug 2013.	Report the association between increasing equity in distribution of health expenditure, and pertinent improvement of equity in the use of services and financial protection.
8	McKee M et al. (18). Universal Health Coverage: A quest for all countries but under threat in some.	Value in Health 2013	Identify five factors which enable the development of UHC: strength of organized labor, availability of resources, building shared identities, path dependency and windows of opportunity.
9	Gera R et al. (19). Sustainable development goals: Leveraging the Global agenda for driving health policy reforms and achieving Universal health coverage in India.	Indian J Community Med Oct-Dec 2018	Opine that unless the system provides adequate financial protection against health expenditure, the poverty alleviation programs in India cannot achieve their targets.
10	Baltussen R et al. (20). Priority setting for Universal health coverage: we need evidence-informed deliberative process, not just more evidence on cost-effectiveness.	Int J Health Policy Manag 2016	Interpret cost-effectiveness in the context of other stakeholder values, where-in the analysis captures the financial protection offered by the interventions to the target population.
11	Prinja S et al. (23). A composite indicator to measure universal health care coverage in India: way forward for post-2015 health system performance monitoring framework.	Health policy and planning 2017	Report a methodology towards computing a composite indicator for measuring the extent of UHC in terms of service coverage, financial risk protection and distributional aspects of service coverage.
12	Marten R et al. (24). An assessment of progress towards universal health coverage in Brazil, Russia, India, China and South Africa (BRICS).	Lancet 2014	Review progress of UHC in BRICS countries and report the relevant issues: insufficient public spending, ensuring equity, managing the needs for logistic support and human resources, stewarding the mixed private and public health systems, managing the burden of diseases and addressing the social determinants of health.

### Framework stage 5: collating, summarizing and reporting the results

The report (17) informs that the system for UHC will cover all sections of the Indian population including the lower socio-economic class, marginalized and hard-to-reach groups, which is addressed in the principle of ‘Universality’. Even the high income groups will benefit from this efficient and equitable health system. The ‘Equity’ principle will enable access to health services, thus improvising the disparity in exposure and vulnerability to diseases among the disadvantaged groups in the community. UHC will promote ‘Empowerment’ of communities towards making better health choices. The ‘Comprehensiveness’ framework will make provision for the maximum range of necessary medical services. ‘Non-exclusion and non-discrimination’ and ‘financial risk protection’ principles address the mitigation of precluding socio-economic factors and pre-existing health conditions, following the provision of services under UHC. The services are delivered in concurrence to the universally accepted standards for ‘patient care and rights’. ‘Continuity of care’ is assured for migrant workers, beneficiaries who change employers or become unemployed, and those with any health insurance coverage. UHC system creates a platform for ‘integrating’ public facilities such as Employee State Insurance scheme hospitals and facilities associated with public agencies like Railways, as well as the regulation of private healthcare providers. The effectiveness of the health system will be periodically reviewed through ‘participatory monitoring’ by stakeholders viz. civil society organizations, public health officials, community representatives and health rights groups. The concepts related to these 10 principles are postulated in the following review:

### Equity

This can be achieved by equitable spread of health care facilities and services, and availability of timely transportation from the underserved areas. WHO has set a benchmark of minimum 445 healthcare personnel/100,000 population. However during the year 2016, India had only 169 Physicians, Nurses and midwifery personnel (including AYUSH doctors but excluding Auxiliary Nurse Midwives) (2). The proportion of health workers/10,000 population in the urban areas is 4 times more when compared with the rural areas (17). In rural areas, 42% of health workers identifying themselves as ‘allopathic doctors’ tend to have no medical training relative to 15% in the urban areas (17). The bias in health financing is reflected by the allocation made to urban allopathic services which is ~30% of the public health expenditure when compared with <12% for the rural centers (17).

The inequities in the Indian healthcare scenario could be attributed to the disregard for evidence based practices in primary healthcare, and the social determinants of health. Certain factors such as social class, gender, religion, caste, urban/rural residence and personal background determine the access to healthcare and quality of services received by the community. Kutzin (18) report the association between increasing equity in the distribution of health expenditure, and pertinent improvement of equity in the use of services and financial protection. The focus of UHC system includes investments in the primary care networks, and holding the Providers accountable for wellness outcomes at the population level. This aim supercedes the existing focus of current insurance schemes on hospital networks and their disregard for primary care services (10).

The response of the public sector system to the needs of the community has been poor, and UHC has the potential to reconfigure the norms of healthcare by ensuring universal reach, quality and accessibility of healthcare services. Under UHC, certain pre-existing health conditions such as HIV/AIDS or the requirement of health service for occupational illness (special category) will not preclude the accessibility of health services. Healthcare services should include the needs of special groups such as on long-term medication support, children with special needs, victims of road traffic accidents and people requiring long-term rehabilitation (3). The persisting challenges in implementation of the scheme includes skewed distribution of public/private hospitals and qualified healthcare providers, high cost of medicines, non-coverage of ambulatory care and lack of awareness among enrollees regarding the empanelled hospitals and eligible conditions (3).

### Coverage

The deliberative process on UHC imbibes blanket criteria which include efficiency of services, cost-effectiveness, necessity and feasibility. Kutzin (18) report the four generic functions of all health systems: stewardship, generating resources (human and physical), service delivery and financing. Covering the population includes provisioning competent healthcare providers who will function from an appropriate infrastructure, with rationing of adequate logistic support including essential medicines, functional equipment, laboratory supplies, other medical needs and transportation facilities. The author (18) informs that the aim of UHC needs to be disaggregated into component objectives which include progress in the following areas: equity in the use of needed health services, quality of service, accountability to the population, administration of the health system and financial protection. The scheme should facilitate seamless healthcare provision during inter-agency referral of insurance coverage, including assistance for transportation of the patient.

The nature of private health sector in India has changed from individual practitioners operating from clinics and nursing homes, to an organized industry attracting huge investments (2). These changes are a result of Health Policy 2002, which recommends private sector participation at the levels of primary, secondary and tertiary care (2). The integration of private health sector into the overall healthcare system needs to be periodically monitored by performance monitoring systems, which include various levels of process and outcome indicators. In this regard, an advanced electronic health record system enables the integration of information. Apart from the strategies for clinical areas, the blending process also aligns along the domains of administration, organization and service delivery. This merging is liaised through standardized protocols and streamlined communication, and entitles an improved quality of performance of the private sector. The Clinical Establishment Act 2010 does not seem to have a large impact on the regulation of the private health sector (2). Their focus on curative services contributes to the curative/treatment paradigm rather than a preventive healthcare approach. The growth of this for-profit private healthcare sector results in increase in health expenditure.

Through diverse public-private partnerships (PPP) schemes, the National Health Policy 2017 intends to address the deficiencies in resources, workforce and management capacity. Sundari et al. (2) report the evidence for PPPs’ which portray neither an increase in the available range of services nor a significant population-level impact on expanding coverage to the impoverished population. This strategy appears to contravene the progress towards UHC. In the State of Karnataka, the PPP for primary healthcare ‘Arogya bandhu scheme’ was scrapped by the State Government during January 2016. The private entities managing the public sector were alleged with non-compliance to regulations, misappropriation of funds, lack of accountability, non-availability of qualified human resource and failure to provide quality service to the patients. The PPP for a tertiary care hospital in Raichur district, Karnataka, where-in the Government provided land, supply of utilities and financial aid to the private Institute was also abruptly terminated. The reasons include poor governance, lack of accountability and patient grievance redressal mechanisms. Also due to the outsourcing involved with PPP, the contractual workforce tends to cope with poor working conditions including lower pay scales (19).

### Financial risk protection

Towards achieving UHC, the four key financing strategies identified by WHO include increasing efficiency of taxation, increasing the health budget of Government, innovation in financing for health sector and providing developmental assistance for health (3). The concept of health financing for UHC includes reforms in collection, pooling, purchasing and designing the benefits (18). The primary strategy of policy makers towards achieving UHC includes publicly financed health insurance scheme (PFHIS) for the low-income population. Zodpey et al. (3) infer from available evidence that the tax revenue is a key determinant in the progress towards UHC in LMICs. The cornerstone of financing the UHC include pre-payment from sources of taxation, and the pooling of such revenue for purchasing healthcare services on behalf of the entire population. UHC enables financial protection of every individual for accessing emergency or essential health care, which includes cashless service at the point of service provision.

Sundari et al. (2) analyzed the earlier PFHIS in India ‘RSBY scheme’, and report that BPL beneficiaries faced difficulties in accessing care from empanelled hospitals. This includes the distance travelled from home, certain health conditions not covered in the benefits package and empanelled hospitals charging some components of the treatment. Another reason includes the low level of financial coverage of Rs.30,000 per family per annum (2). The authors (2) infer that the unresolved problems of AB-PMJAY include low budgetary allocation by the Government and inequalities in access and utilization, which hinders its benefaction to UHC. Additional evidence on PFHS infers the paucity in addressing the out-of-pocket expenditure among the enrolled households, following its implementation (20). Kutzin (18) report that polarized financing arrangements will result in a 4.5 to 6 times higher total expenditure for the insured population, when compared with people using PFHS. The social health insurance (SHI) scheme for the formal sector employees are skewed towards the economically advantaged, and paralyzed by an inequality in entitlement of explicit healthcare services (e.g., hemodialysis treatment, renal replacement therapy) (18).

Reddy K.S (8) et al. opine that public spending on health should be increased from ~1 to 6% of the GDP. This will enable reducing the proportion of out-of-pocket expenditure from 80 to 20%. The authors (8) opine that the investment on health research should be increased to 8% of the health budget. The High level expert group (17) recommends an increase in the public procurement of medicines from ~0.1 to 0.5% of GDP. The health system measures which reduce financial barriers for using the services are likely to increase its utilization across the entire population. The UHC related insurance scheme will enable gains in system-wide efficiency, and foster equity for both the formal and informal sector population.

### Efficiency

One of the goals of the health system is to make better utilization of the available resources. The progress towards UHC enables a shift in the focus from insurance schemes to the health system. This should be measured in-terms of the impact of financing policies on the quality of health service, equity in its usage and financial protection of the entire population, rather than the proportion of population covered by the scheme. WHO has advised the three most essential criteria for countries, while setting priorities for UHC which include cost effectiveness, priority to the worse-off and financial protection (21). This enables transparent decision making while using multiple metrics, and bestow relevance to social values (3). Baltussen et al. (21) discuss the interpretation of cost-effectiveness in the context of other stakeholder values. Certain countries with financial reforms for universal protection tend to have shortfalls in effective coverage (18), which re-affirms the shift in criterion from achieving UHC to moving towards UHC.

The public healthcare sector is plagued with manpower issues including lack of innovation and harmony within the organization. The recruitment initiatives for such positions should promote adequate incentives, progression through the career track and distinctive work environment. The creation of a cadre of Indian National Health Services in the Public sector rather than the existing general administrators will enable public health personnel in planning and monitoring health program implementation. The effective implementation of UHC will be facilitated by the efficient planning of comprehensive primary health care programs (22). Strategies and interventions focused on health systems will enable improvement in quality (23). Zodpey et al. (3) infer that an UHC based health system could efficiently regulate the complex and dynamic private health sector, as it generates market choices which in-turn has the potential to enhance quality of care and reduce the cost of care. The complex processes in healthcare need to be effectively managed using systems which will enable coordination of multiple resources and diverse communities.

### Transparency and accountability

People should be aware of the variety of entitled services, which will empower them to demand the essential health services. The public domain will provide general information regarding the functioning of the system, and certain specific information will be provisioned through the Right to information act. Transparency thus induced will enable reducing the gap between need for services and their utilization. This will enable rooting out the existing corruption and malpractices, and punitive action against offenders will further deter such attempts. Kutzin (18) informs that lack of transparency might lead to informal payments, resulting in curtailed financial protection. Such experiences reinforce the need for a decentralized governance structure which responds to the local needs of the community.

A strong political will ensures that investment for public welfare is closely associated with economic growth. On the contrary, privatized health care delivery and finance will enable profits to the strong alliance of private insurance companies, pharmaceutical companies and medical associations. Health financing agencies which use public resources should establish accountability, which in-turn translates to the better use of resources. The empowered civil society groups will entitle the creation of systems for accountability of healthcare, including community participation in health planning and operationalizing grievance redressal mechanisms. Organizations such as village health sanitation and nutrition committees have certain operational deficits due to their low proficiency and ambiguity of roles. These gaps should be addressed through sustained training for strengthening their capacities (24). The scheme facilitates protection of patients’ rights including: right to confidentiality and privacy, right to emergency medical care, right to information, right to informed consent, right to second opinion, right to choose between treatment options including refusal of treatment.

### Policy initiatives

The focus of UN health organizations and Rockefeller Foundation has been on the quartet of interventions: growth monitoring, oral rehydration, breast feeding and immunization. This dissipates the distinct Alma-ata declaration of 1978, which prioritizes the principle of universal primary health care. Although these interventions enabled the gains in specific domains of health, the evolving epidemics of HIV and NCDs (non-communicable disease) however reflect the disparate needs of the population and health systems. The Millenium Development Goals (25) adopted during the year 2000, provides the stimulus for expanding health service provision in LMICs. Kumar et al. (10) propose the establishment of an autonomous public health commission in India, which will enable achieving the coordination of initiatives such as health promotion, disease prevention and provision of health care. Health policies and systems should not be enforced solely as a ‘State subject’, but ideally as a concurrent subject of both the Central and the State Governments.

Mckee et al. (20) identify five factors which enable the development of UHC: strength of organized labor, availability of resources, building shared identities, path dependency and windows of opportunity. The study mentions the lack of strong labor movement in LMICs unlike the industrialized nations, which is due to the macroeconomic policy of foreign investment promising low-wages and a union-free environment. Certain vested interests ingrained in the existing healthcare system which is primarily reliant on the private finances, in-turn could hamper the future role of public sector in the provision of healthcare including its finances. The concept of path dependency exists when the present circumstances and past choices tend to shape the future path.

### Current pandemic scenario

Chi et al. (13) enumerate the Covid-19 related channels which could exacerbate the constraints on public health financing in LMICs. These encompass the decline of GDP which diminishes the Government revenues, the reduction in support from external sources and the additional health costs due to covid response. A waning of domestic/international trade will result in declining revenues from consumption taxes (e.g., Goods and Sales Tax). As economies of high income Countries (HIC) dwindle, their support to LMIC for managing covid pandemic will decline. This has influence on other health sectors such as the fight against HIV/AIDS, where-in half the resources spent by LMIC are contributed by the external sources. The covid response including testing facilities, tracing systems, treatment costs and other logistics demand exceptional allocation of budget. As the Covid-19 vaccination coverage is extended beyond vulnerable age groups, the expenditure on meeting vaccination target will represent multiples of our Country’s health budget.

The pandemic has provided us an opportunity to reform tax systems in ways which could benefit UHC, including Provider payments for the provision of essential services and de-listing UHC interventions which are not cost-effective (26). Among other health priorities, the surveillance systems should be upgraded as per the report of WHO’s SCORE (24) initiative. This enables tracking of COVID patients and contacts, progress of COVID vaccination campaigns and recording of the related deaths.

### Evaluation

Prinja et al. (27) report a methodology for computing a composite indicator towards measuring the extent of UHC, which is similar to the framework proposed by WHO and World Bank. This includes service coverage, financial risk protection and distributional aspects of service coverage. Beyond the WHO framework, the aspects of quality of care computed include curative care from a qualified healthcare provider, complete and effective antenatal care as well as the met need of curative care for NCDs. However, the lack of reliable data limits the inclusion of services such as rehabilitation, palliation or long-term care coverage. Also, the measurement matrix did not incorporate services related to the social determinants of health.

For measuring the financial risk protection, 2 indicators used to assess ‘depth of poverty’ include (27):

- worsening of the household’s existing level of poverty due to the out-of-pocket health payments,
- ‘mean catastrophic positive overshoot’: health related payment more than the threshold used to define catastrophic health spending,

The measurement of progress in a health financing scheme needs a systems approach, where-in the impact on equity in usage of health services and financial protection across the entire population takes precedence over the percent of population covered by the scheme (18).

## Discussion

The scoping review marginally synthesizes findings from varied study designs, unlike systematic reviews which tend to focus on randomized controlled trials (single study design) (15). This study maps the relevant literature regarding UHC, its deliverables, policy formulation and strategies for improvisation. UHC aims to address the concept of health in all its dimensions and not merely as a response to illness. Its objectives include reducing the gap between need and utilization, improving the quality of care and providing financial protection (18). The process includes a direction driven approach rather than a destination one. The progress of UHC in India involves repositioning the existing health systems, presently facilitated by the integration of SDG agenda in NHP-2017 (7) and NITI (National Institution for Transforming India) Aayog’s (28) Vision for Health 2032.

The universality concept of UHC is a social need as a large section of the Indian population including the middle class, lacks access to affordable and quality healthcare. The gaps in the healthcare delivery system of India cannot solely be bridged by additional investment, hiring of manpower, better technology and regulatory Institutions. Zodpey et al. (3) infer that it is critical for the AB-PMJAY to ensure the gatekeeping function of the insurance regulators, given the experience of the earlier RSBY scheme. This enables addressing the moral hazard between the supply and demand sides, which is the hallmark of insurance-driven schemes (6). The quality of care rendered at Health and Wellness centers should be evaluated through a medical audit done as per the defined standards.

As per the 2019 UN Human Development Report (29), 27.9% of India’s population is multi-dimensionally poor. Gera et al. (30) opine that unless the administrative system provides adequate financial protection against health expenditure, the poverty alleviation programs cannot achieve their targets. Health services need to be financed by pre-payments, such as health insurance schemes or Government tax revenues (3). The tax based financing should be supplemented by a unique SHI scheme for employees of the formal sector, which involves a payroll deduction for all salaried employees matched by their employer’s contribution. Self-employed persons need to pay a fixed amount as insurance premium, and the poor are subsidized or completely paid by the Government. Evidence shows that UHC cannot be achieved through voluntary contribution or small group insurance schemes (18).

In the past, policy reforms for low-income households have focused on expanding the role of private sector and publicly financed health insurance schemes. The UHC based health system should efficiently regulate the complex and dynamic nature of the private healthcare sector. The market competition and choices generated therewith should be used as tools to enhance the quality of care and diminish the costs. A pluralistic UHC driven healthcare system should enable the engagement of multiple stakeholders, given that the social determinants of health influence the equitable distribution of healthcare. Scattered sections of the population will need unique health services which could be ensured as ‘vertical equity’ in the UHC framework. Other innovative policies include financial and non-financial incentives for health workers in remote areas, and compulsory 1 year rural service for newly graduating Doctors, Dentists, Nurses and Pharmacists, which albeit conceals the Government’s deficiency in developing rural health systems.

At the point of care, the range of healthcare services should be comprehensive and made available at no cost (out-of-pocket payment aside), thus covering both inpatient and outpatient care including the cost of drugs. Digital health is purported to bring healthcare within the reach of 70% of our population (2). Other areas which need to be integrated include safe water supply, sanitation, nutrition and healthy lifestyle. The deliberations on UHC in the past have been dominated by focusing on clinical services, which are targeted at individuals. In contrast to clinical services, public health interventions engender different dynamics for generating evidence towards informed policy making. The study summarizes and disseminates research findings related to UHC, but does not appraise the quality of evidence in the primary research reports.

The strength of our scoping review includes an astute synthesis of information about UHC in India, which conduces the framing of policy decisions by the health authorities. It maps the available resources, and addresses broad research concepts concerning the implementation of UHC in India. However the limitation of this review includes a dearth of comprehensive search strategy, given the emerging nature of the topic. Also the paucity of resources such as supporting data, and web based tools namely Rayyan, Covidence or NVivo. The utilization of such assets would enable an assessment of bias in evidence and defining the boundaries for implementation of UHC.

This review can forbode other empirical enquiries about UHC in India, which includes evidence based systematic reviews. Apart from the issues deliberated in this article, innovative strategies should focus on approaches such as social/environmental determinants of ill-health, streamlining Center-State linkages, sanctioning an autonomous public health commission, decentralization of health planning and monitoring, broadening the insurance coverage, conforming costs to public budget and use of appropriate technology. Future research should focus on health system preparedness for emerging health threats, given the changing ecosystems.

## Conclusion

Progress towards UHC involves working through the health systems, and not just the financial investments. In this regard, certain intermediate objectives include improving the efficiency of healthcare services, equity in the distribution of resources, accountability and financial protection. This will ensure provision of health care for all including vulnerable individuals from poor or rural background, children and age group. In the Indian context, UHC needs to be financed by increasing the tax-based public financing as we have a low proportion of formal sector employees. The way forward is to develop a comprehensive integrated health insurance service which is financed through a combination of public sector funds, occupational coverage and private sources. Future research should include ‘Consultation exercise’ as a framework stage (15) for stakeholder engagement. In view of the current Covid-19 pandemic and the need to address future pandemics, we need to build resilient health systems as well as hasten the implementation of UHC.

## References

[1] Lauer JA Rajan D Bertram MY. Priority setting for universal health coverage: we need to focus both on substance and on process. Int J Health Policy Manag. (2017) 6:601–3. doi: 10.15171/ijhpm.2017.06, PMID: 28949475 PMC5627787

[2] Sundari TK Neena EP. Towards universal health coverage? Taking stock of two decades of health reforms in India. Indias Econ Soc. (2021):253–85. doi: 10.1007/978-981-16-0869-8_10, PMID: 39687769

[3] Zodpey S Farooqui HH. Universal health coverage in India: progress achieved & the way forward. Indian J Med Res. (2018) 147:327–9. doi: 10.4103/ijmr.IJMR_616_18, PMID: 29998865 PMC6057252

[4] Reddy KS Patel V Jha P Paul VK Kumar AKS Dandona L. Towards achievement of universal health care in India by 2020: a call to action. Lancet. (2011) 377:760–8. doi: 10.1016/S0140-6736(10)61960-5, PMID: 21227489 PMC4991755

[5] High level Expert group report on Universal health coverage for India (2011). Submitted to the planning Commission of India, New Delhi. Available at: http://www.niti.gov.in/planningcommission.gov.in/docs/reports/genrep/rep_uhc0812.pdf, (Accessed August 2021).

[6] National Health Profile (2019). Available at: http://www.cbhidghs.nic.in/showfile.php?lid=1147 (Accessed August 12, 2021).

[7] National Health Policy (2017). Available at: www.nhp.gov.in/nhpfiles/national_health_ policy_2017.pdf (Accessed February 5, 2022).

[8] Rashtriya Swasthya Bima Yojna (2021). Available at: india.gov.in/spotlight/rashtriya-swasthya-bima-yojana (Accessed February 5, 2022).

[9] Authors (2022). Number of towns, villages, households, population and area. Available at: https://censusindia.gov.in/census.website/data/census-tables (Accessed September 14, 2022).

[10] Kumar R. Achieving universal health coverage in India: the need for multisectoral public health action. Indian J Community Med. (2020) 45:1–2. doi: 10.4103/ijcm.IJCM_61_19, PMID: 32029973 PMC6985948

[11] National Health Mission (2022). Available at: nhm.gov.in (Accessed February 5, 2022).

[12] Ayushman Bharat Pradhan Mantri Jan Arogya Yojana (2021). Available at: https://nha.gov.in/PM-JAY (Accessed February 5, 2022).

[13] Socio Economic and Caste Census 2011 (2012). Available at: www.secc.gov.in (Accessed February 5, 2022).

[14] National Health Authority (2018). Available at: pmjay.gov.in (Accessed June 10, 2021).

[15] Arksey H O’Malley L. Scoping studies: towards a methodological framework. Int J Soc Res Methodol. (2005) 8:19–32. doi: 10.1080/1364557032000119616

[16] Tricco AC Lillie E Zarin W O'Brien KK Colquhoun H Levac D . PRISMA extension for scoping reviews (PRISMAScR): checklist and explanation. Ann Intern Med. (2018) 169:467–73. doi: 10.7326/M18-0850, PMID: 30178033

[17] High level Expert group report on Universal health coverage for India (2011). Submitted to the planning Commission of India, New Delhi. Available at: http://www.niti.gov.in/planningcommission.gov.in/docs/reports/genrep/rep_uhc0812.pdf, (Accessed August 30, 2022).

[18] Joseph K. Health financing for universal coverage and health system performance: concepts and implications for policy. Bull World Health Organ. (2013) 91:602–11. doi: 10.2471/BLT.12.113985, PMID: 23940408 PMC3738310

[19] Belagere C. (2020). Public-private partnership in healthcare not good idea: Experts. Available at: http://www.newindianexpress.com/nation/2020/jan/03/public-private-partnership-in-healthcare-not-good-idea-experts-2084375.html (Accessed August 30, 2022).

[20] McKee M Balabanova D Basu S Ricciardi W Stuckler D. Universal health coverage: a quest for all countries but under threat in some. Value Health. (2013) 16:S39–45. doi: 10.1016/j.jval.2012.10.001, PMID: 23317643

[21] Baltussen R Jansen MP Mikkelsen E Tromp N Hontelez J Bijlmakers L . Priority setting for universal health coverage: we need evidence-informed deliberative process, not just more evidence on cost-effectiveness. Int J Health Policy Manag. (2016) 5:615–8. doi: 10.15171/ijhpm.2016.83, PMID: 27801355 PMC5088720

[22] Jayanna K Swaroop N Kar A Ramanaik S Pati MK Pujar A. Designing a comprehensive non-communicable diseases (NCD) programme for hypertension and diabetes at primary health care level: evidence and experience from urban Karnataka, South India. BMC Public Health. (2019) 19:409. doi: 10.1186/s12889-019-6735-z, PMID: 30991978 PMC6469122

[23] Jayanna K. Improving quality of care in maternal, newborn and child health: opportunities and challenges for India. Indian J Community Health. (2013) 25:327–9.

[24] Potty RS Lakkappa MH Kar A Bidappa M Manjappa RB Jayanna K. Influence of integrated community and facility based interventions on select maternal and neonatal outcomes in northern Karnataka, India: lessons for implementation and measurement. Indian J Public Health. (2017) 61:19–25. doi: 10.4103/0019-557X.200256, PMID: 28218158

[25] Authors (2017). Milleniium development goals and beyond 2015. Available at: www.un.org/milleniumgoals/ (Accessed February 5, 2022).

[26] Chi L.Y Glassman A Ghosh S Regan L. (2021). How will Covid-19 impact our progress towards universal health coverage? Available at: www.cgdev.org/blog dt:10/2/21. (Accessed June 10, 2022).

[27] Prinja S Gupta R Bahuguna P. A composite indicator to measure universal health care coverage in India: way forward for post-2015 health system performance monitoring framework. Health Policy Plan. (2017) 32:43–56. doi: 10.1093/heapol/czw097, PMID: 27497138

[28] NITI Aayog (2022). Available at: www.niti.gov.in (Accessed February 6, 2022).

[29] Human Development Report (2019). Available at: www.hdr.undp.org/sites/default/files/hdr2019.pdf (Accessed February 4, 2022).

[30] Gera R Narwal R Jain M. Sustainable development goals: leveraging the global agenda for driving health policy reforms and achieving universal health coverage in India. Indian J Community Med. (2018) 43:255. doi: 10.4103/ijcm.IJCM_41_18, PMID: 30662175 PMC6319280

[31] Authors (2012). Monthly income of highest earning household member in all category households. Available at: https://secc.gov.in/getAllCategoryIncomeSlabNationalReport.htm (Accessed September 15, 2022).

